# Predictive value of PD-L1 and TMB for short-term efficacy prognosis in non-small cell lung cancer and construction of prediction models

**DOI:** 10.3389/fonc.2024.1342262

**Published:** 2024-05-02

**Authors:** Shuling Shi, Yingyi Wang, Jingjing Wu, Boya Zha, Peihong Li, Yukun Liu, Yuchuan Yang, Jinglin Kong, Shibo Gao, Haiyang Cui, Linkuan Huangfu, Xiaocong Sun, Zhikai Li, Tiansong Liang, Yingjuan Zheng, Daoke Yang

**Affiliations:** ^1^ Department of Radiation Oncology, The First Affiliated Hospital of Zhengzhou University, Zhengzhou, Henan, China; ^2^ Institute of Radiotherapy and Critical Care Oncology, Zhengzhou University, Zhengzhou, Henan, China

**Keywords:** non-small cell lung cancer, PD-L1, tumor mutation burden, objective response rate, nomogram model

## Abstract

**Objective:**

To investigate the correlation between programmed death ligand 1(PD-L1), tumor mutation burden (TMB) and the short-term efficacy and clinical characteristics of anti-PD-1 immune checkpoint inhibitor combination chemotherapy in NSCLC patients. The efficacy of the prediction model was evaluated.

**Methods:**

A total of 220 NSCLC patients receiving first-line treatment with anti-PD-1 immune checkpoint inhibitor combined with chemotherapy were retrospectively collected. The primary endpoint was short-term efficacy ORR. The correlation between short-term efficacy, PD-L1, TMB, and clinical characteristics using χ2 test or t-test was evaluated. Screen the independent prognostic factors using univariate and multivariate logistic regression analyses, and construct a nomogram prediction model using the “rms” package in R software. Using receiver operating characteristic (ROC) curve analysis to evaluate the independent Prognostic factors and the prediction model. Using decision curve analysis (DCA) to verify the superiority of the prediction model.

**Results:**

The mean values of PD-L1, TMB, neutrophils, lymphocytes, neutrophil-to-lymphocyte ratio, and albumin were the highest in the ORR group, PD-L1 expression and TMB correlated with epidermal growth factor receptor expression. Multivariate analyses showed that PD-L1, TMB, and neutrophil were independent prognostic factors for ORR. The area under the ROC curve (AUC) values of the ROC constructed based on these three indicators were 0.7104, 0.7139, and 0.7131, respectively. The AUC value under the ROC of the nomogram model was 0.813. The DCA of the model showed that all three indicators used together to build the prediction model of the net return were higher than those of the single indicator prediction model.

**Conclusion:**

PD-L1, TMB, and neutrophils are independent prognostic factors for short-term efficacy. The nomogram prediction model constructed using these three indicators can further improve predictive efficacy of ICIs in patients with NSCLC.

## 1 Introduction

Lung cancer is the leading cause of cancer-related deaths, and non-small cell lung cancer (NSCLC) is the most common type of lung cancer. Most lung cancer patients are diagnosed when the disease has progressed to the middle or late stages; however, traditional chemoradiotherapy has a low cure rate and high adverse effects, and the 5-year survival rate is less than 22% (1). Therefore, new therapeutic modalities are urgently needed to improve disease control and prolong OS in patients with lung cancer.

Among new therapeutic modalities, targeted therapies have the best efficacy for NSCLC patients, especially those carrying epidermal growth factor receptor (EGFR) mutations (2, 3), However, targeted therapies inevitably generate drug resistance, posing a challenge for the follow-up of NSCLC patients. Meanwhile, immunotherapy using immune checkpoint inhibitors (ICI) has brought new hope for patients with Solid tumor. ICIs are based on the tumor cell immune escape (4)mechanism which regulates the immune microenvironment around tumor cells and restores their antitumor function of immune cells (5). ICIs have been applied to various cancers, such as melanoma, renal cell carcinoma, bladder cancer, and mismatch repair-deficient solid tumors (6–9), and have achieved good clinical efficacy.

The main ICIs currently in clinical use for NSCLC are anti- PD-1/PD-L1 ICIs. PD-1 and PD-L1 are usually highly expressed on the surface of activated lymphocytes and tumor cells, respectively, and the combination of the two down-regulates TCR signaling and reduces the production of TNF-α, IFN-γ, and IL-2 (10), which inhibits the tumor-killing function of immune cells. Anti-PD-1/PD-L1 ICIs are novel therapeutic modalities that restore immune killing functions by blocking this pathway. A large number of studies have confirmed that anti-PD-1/PD-L1 ICIs produce better clinical therapeutic effects in patients with NSCLC, increasing the objective response and survival rates, and improving the quality of patient survival (11, 12).

According to the 2023 CSCO guidelines for the diagnosis and treatment of NSCLC, ICIs have been used as first- and second-line treatments for advanced NSCLC. However, as ICIs are widely used in patients with NSCLC, the resistance and immune-related adverse events (irAEs) they generate have become the main factors limiting the clinical application of ICIs (13). In this context, finding accurate predictive markers has become the main research focus to solve this problem.

At present, PD-1/PD-L1 expression levels, and tumor mutation burden have become the main detection biomarkers before clinical immunotherapy, with the aim of screening patients with NSCLC who are suitable for individualized immunotherapy. However, for many patients with advanced NSCLC, obtaining sufficient tumor samples is a significant limiting factor. Therefore, this study aimed to investigate the predictive value of PD-L1 and TMB on the short-term efficacy of first-line treatment with ICIs in combination with chemotherapy in patients with locally advanced NSCLC and to provide a theoretical basis for the clinical development of individualized treatment.

## 2 Materials and methods

### 2.1 Clinical data

This study retrospectively collected data from 220 patients with NSCLC who visited the First Affiliated Hospital of Zhengzhou University from September 2021 to September 2023 and received first-line treatment with anti-PD-1 ICIs combined with chemotherapy. The inclusion criteria were as follows: (1) pathological type of NSCLC; (2) stage III or IV according to TNM staging; (3) patients with primary diagnosis; (4) patients receiving anti-PD-1 immune checkpoint inhibitor therapy as first-line treatment; (5) patients with KPS score >70 before treatment; (6) patients aged 18–80 years (7) with at least one measurable lesion. The exclusion criteria were as follows: (1) previous history of malignant tumors or current diagnosis of dual primary tumors; (2) incomplete clinical data; and (3) combined immune-related diseases. All patients underwent complete blood routine, liver and kidney function, chest MRI/CT abdominal CT, brain MRI, whole-body bone ECT, and other related examinations. This study was approved by the Ethics Committee of the First Affiliated Hospital of Zhengzhou University.

### 2.2 Treatment

All the patients are treated with a chemotherapy + anti-PD-1 immune checkpoint inhibitor regimen. nab-PP regimen was adopted for synchronous chemotherapy (albumin paclitaxel 100 mg/m^2^, ivgtt d1, 8, 15 + cisplatin 75 mg/m^2^/carboplatin area under the receiver operating characteristic (ROC) curve (AUC) = 5-6, ivgtt d1), AP regimen (pemetrexed 500 mg/m^2^, ivgtt d1 + cisplatin 75 mg/m^2^, ivgtt d1/carboplatin AUC = 5–6, ivgtt d1), GP regimen (gemcitabine 1,000 mg/m^2^, ivgtt d1,8 + nedaplatin 80 mg/m^2^ ivgtt d1), DP regimen (docetaxel 70 mg/m^2^ ivgtt d1 + carboplatin AUC = 5–6, ivgtt d1). Immunotherapy was administered via an intravenous drip of camrelizumab/sintilimab/tislelizumab/pembrolizumab 200 mg. The first day was used, and the course of treatment was 21 days. Some patients were followed-up with immune checkpoint inhibitor consolidation therapy.

### 2.3 Indicators

Immunohistochemical PD-L1 detection index: specimen, tumor tissue section of the patient; detection reagent, monoclonal mouse anti-human PD-L1; evaluation method: TPS, number of PD-L1 staining positive tumor cells/total number of live tumor cells * 100%; cut-off value for judging the test results: <1% was negative, 1%–49% was low expression, and ≥50% was high expression. TMB was defined as the total number of somatic mutations detected per million bases (muts per Mb unit, muts/Mb). Specimen: tumor tissue FFPE; detection reagent: DNA extraction kit (OMEGA); Detection method: DNA-based probe capture library building method high-throughput sequencing (NGS); evaluation method: according to the current clinical research data, TMB > 10 muts/Mb was considered high. The absolute value of neutrophils (Neut) was defined as the number of neutrophils in the white blood cell count, with a normal reference range of 1.8 − 6.3 * 10 ^ 9/L. The absolute value of lymphocytes (LYM) refers to the specific number of lymphocytes in the blood, with a normal reference range of 1.1 − 3.2 * 10 ^ 9/L. Neutrophil-to-lymphocyte ratio (NLR) was defined as the ratio of routine neutrophil-to-lymphocyte counts. Albumin (Alb), a major protein in human plasma, was also used as an indicator.

### 2.4 the evaluation of curative effect

All first-line treatment patients were evaluated for short-term efficacy 3 months after the first course of treatment. The evaluation criteria were response evaluation criteria in solid tumors (RECIST). The evaluation indexes were complete response (CR), partial response (PR), stable disease (SD), and progressive disease (PD). The objective response rate (ORR) was defined as the proportion of patients whose tumor volume shrank to a predetermined value and could be maintained in the minimum time limit, as the sum of the proportion of CR + PR; non-ORR was defined as the sum of the proportion of SD + PD. Efficacy was evaluated every 6 weeks, and CT, MRI, and other related examinations showed improvement.

### 2.5 Statistical processing

**Figure 1:**
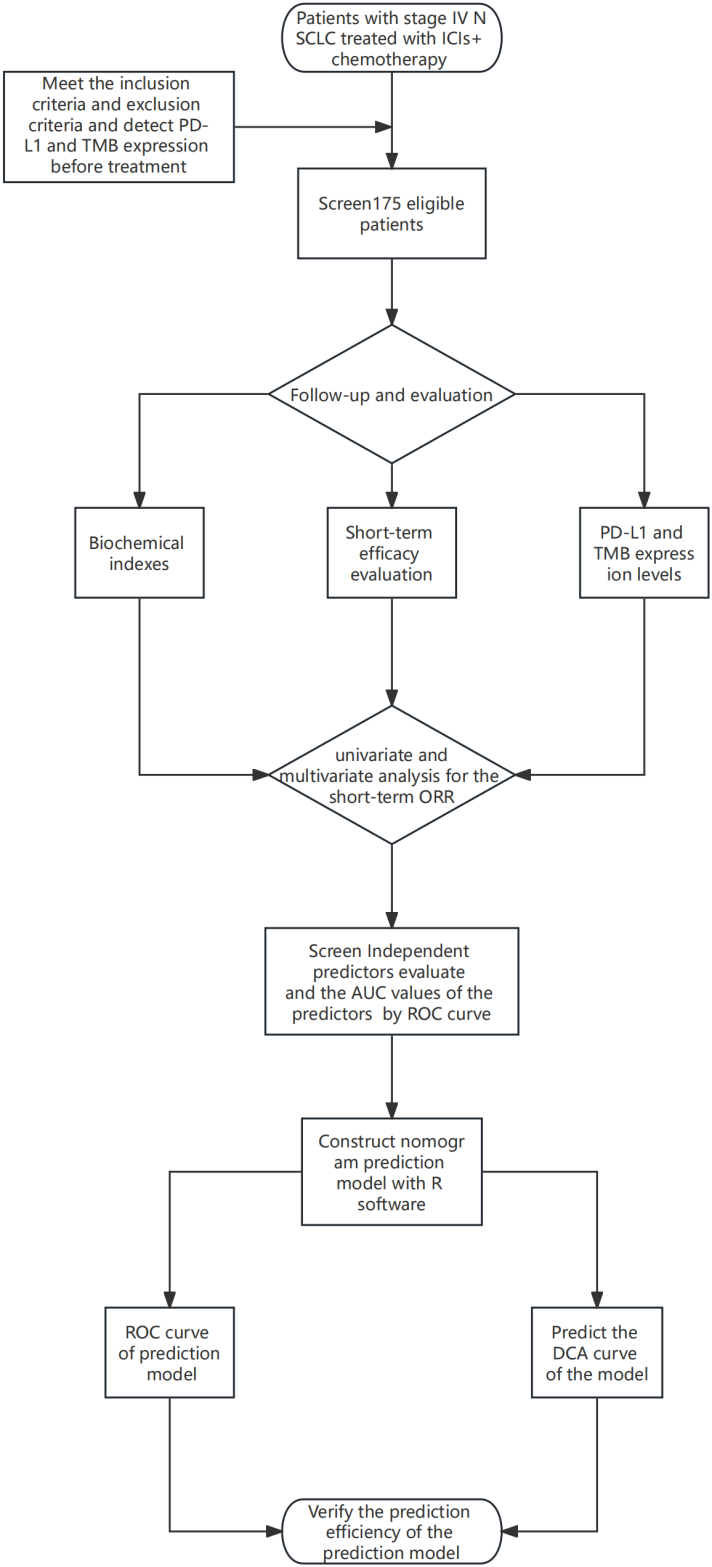
The nomogram construction flowchart.

Statistical analyses were performed using SPSS 23.0, and GraphPad Prism 8 software was used for plotting. The diagnostic efficacy of the different indicators was evaluated using the ROC. Count data were expressed as the number of cases and the rate (%); the χ2 test or Fisher’s exact probability method was used for the qualitative data of the general clinical data and treatment results of the patients included in the study, and the t-test was used for the quantitative data. Prognostic factors were evaluated using univariate and multivariate logistic regression analyses, and the independent prognostic variables in the logistic regression analyses were used to construct a nomogram prediction model using the “rms” package in R software. ROC analysis was used to evaluate the predictive efficacy of the prognostic factors and nomogram prediction model, and decision curve analysis (DCA) was used to verify the superiority of the prediction of this model. the nomogram construction flowchart as shown in **Figure 1**.

## 3 Results

### 3.1 General data

A total of 220 patients were enrolled: 127 in the ORR group and 93 in the non-ORR group. A comparison of the general data of the two groups is presented in **Table 1**. Immunotherapy efficacy was not related to alcohol consumption history, hypertension history, diabetes history, T stage, N stage, M stage, TNM stage. The ORR was the highest in men, age ≥60 years, history of smoking, and patients with squamous carcinoma. The mean values of PD-L1, Neut, LYM, and NLR in the ORR group were greater than those in the non-ORR group, whereas the mean value of Alb was relatively higher in the non-ORR group, and the differences were all statistically significant (*P* < 0.05).

**Table 1:** General data of the two groups of patients.

clinical characteristic	ORR127	non-ORR93	*P*
Sex			<0.001
Man	109	59	
Women	18	34	
age(years)			0.005
≥60	94	52	
<60	33	41	
smoking history			0.008
Yes	65	31	
No	62	62	
Alcohol consumption history			0.250
Yes	36	20	
No	91	73	
Hypertension history			0.646
Yes	32	26	
No	95	67	
Diabetes history			0.156
Yes	19	8	
No	108	85	
T staging			0.388
T1~T2	73	48	
T3~T4	54	45	
N staging			0.649
N0~N2	87	61	
N3	40	32	
M staging			0.636
M0	56	44	
M1	71	49	
TNM staging			0.842
III	57	43	
IV	70	50	
Pathological classification			0.014
squamous carcinoma	69	35	
Adenocarcinoma	58	58	
PD-L1(%)	67.34 ± 0.32	58.02 ± 0.57	<0.001
TMB (muts/Mb)	15.28 ± 0.20	9.59 ± 0.17	<0.001
Neut(*10^9/L)	6.48 ± 0.11	5.15 ± 0.12	<0.001
LYM(*10^9/L)	3.43 ± 0.35	1.78 ± 0.05	<0.001
NLR	8.50 ± 0.44	4.09 ± 0.17	<0.001
Alb(g/L)	39.47 ± 0.08	39.85 ± 0.07	<0.001

### 3.2 Correlation analysis of PD-L1, TMB and clinical features

PD-L1 and TMB were grouped according to clinical study data. PD-L1 high expression group: PD-L1 ≥50%, low expression group: PD-L1 <50%; TMB high expression group: TMB ≥10 muts/Mb; low expression group: TMB <10 muts/Mb. The relationship between PDL1, TMB, and the clinical characteristics of patients with NSCLC is shown in **Table 2**. The PD-L1 score was significantly correlated with EGFR drive type and TMB, whereas the TMB score was correlated with sex, smoking history, drinking history, and T stage.

**Table 2:** Correlation analysis of PD-L1, TMB and clinical features.

clinical characteristic	PD-L1		*P1*	TMB		*P2*
	≥50%	<50%		≥10 muts/Mb	<10 muts/Mb	
Sex			0.141			0.004
Man	64	104		83	85	
Women	14	38		14	38	
age(years)			0.118			0.184
≥60	57	89		69	77	
<60	21	53		28	46	
smoking history			0.158			0.003
Yes	39	57		53	43	
No	39	85		44	80	
Alcohol consumption history			0.488			0.023
Yes	22	34		32	24	
No	56	108		65	99	
Hypertension history			0.436			0.895
Yes	23	35		26	32	
No	55	107		71	91	
Diabetes history			0.540			0.708
Yes	11	16		11	16	
No	67	126		86	107	
T staging			0.067			0.001
T1~T2	44	77		65	56	
T3~T4	34	65		67	32	
N staging			0.458			0.717
N0~N2	50	98		64	84	
N3	28	44		33	39	
M staging			0.471			0.603
M0	38	62		46	54	
M1	40	80		51	69	
TNM staging			0.471			0.804
III	38	62		45	55	
IV	40	80		52	68	
Pathological classification			0.274			0.560
squamous carcinoma	33	71		48	56	
Adenocarcinoma	45	71		49	67	
PD-L1(%)				30.31 ± 3.39	29.96 ± 2.90	0.937
TMB (muts/Mb)	10.81 ± 0.91	8.20 ± 0.43	0.004			
Neut(*10^9/L)	5.28 ± 0.25	4.87 ± 0.19	0.193	5.01 ± 0.17	5.02 ± 0.23	0.967
LYM(*10^9/L)	1.87 ± 0.24	1.54 ± 0.05	0.09	1.81 ± 0.20	1.54 ± 0.06	0.139
NLR	3.88 ± 0.55	3.47 ± 0.20	0.396	3.27 ± 0.23	3.89 ± 0.38	0.186
Alb(g/L)	39.01 ± 0.63	39.12 ± 0.34	0.866	39.58 ± 0.52	38.69 ± 0.38	0.160

### 3.3 Univariate analysis of ORR

The results of the univariate analysis of ORR are shown in **Table 3**. Sex, age, smoking history, pathologic staging, PD-L1, TMB, neutrophils, and NLR were significantly associated with ORR in NSCLC (*P* < 0.05). There were no significant correlations among drinking history, hypertension history, diabetes history, T staging, N staging, M staging, TNM staging, lymphocytes, albumin, or ORR.

**Table 3:** Univariate analysis of ORR in NSCLC patients.

clinical characteristic	B	OR	95%CI	*P*
genders(Man vs Women)	-1.250	0.287	0.149~0.551	<0.001
age(years)(<60 vs≥60)	0.840	2.316	1.306~4.180	0.004
smoking history(yes vs no)	0.740	2.097	1.205~3.649	0.009
Alcohol consumption history(yes vs no)	0.367	1.444	0.771~2.704	0.251
Hypertension history(yes vs no)	-0.142	0.868	0.474~1.589	0.646
Diabetes history(yes vs no)	0.626	1.869	0.780~4.478	0.160
T staging (T1~2 vs T3~4)	-0.237	0.789	0.461~1.351	0.388
N staging(N0~2 vs N3)	-0.132	0.876	0.496~1.547	0.649
M staging(M0 vs M1)	0.130	0.636	0.665~1.948	0.636
TNM staging(IIIvs IV)	0.055	1.056	0.617~1.807	0.842
Pathological classification(squamous carcinoma vs Adenocarcinoma)	-0.679	0.507	0.294~0.875	0.015
PD-L1(%)	0.025	1.026	1.016~1.036	<0.001
TMB (muts/Mb)	0.157	1.170	1.101~1.243	<0.001
Neut(*10^9/L)	1.511	4.533	2.437~8.429	<0.001
LYM(*10^9/L)	0.154	1.166	0.830~1.639	0.375
NLR	0.143	1.154	1.008~1.321	0.038
Alb (g/L)	0.027	0.973	0.918~1.032	0.364

### 3.4 Multivariate analysis results of ORR

The results of the multivariate analysis of ORR are shown in **Table 4**. Eight significant results from the univariate analysis were included in the multivariate analysis. The results showed that PD-L1 expression (*P* < 0.001), TMB (*P* < 0.001), and neutrophils (*P* < 0.001) were independent prognostic factors for ORR in patients with NSCLC. Sex, smoking history, age, pathological classification, and NLR were not independent prognostic factors for the ORR in patients with NSCLC.

**Table 4:** Multifactorial analysis of ORR in NSCLC patients.

clinical characteristic	B	OR	95%CI	*P*
sex(Man vs Women)	-0.250	0.779	0.309~1.964	0.596
smoking history(yes vs no)	-0.080	0.923	0.423~2.013	0.923
age(years)(<60 vs≥60)	0.448	1.565	0.768~3.189	0.218
Pathological classification(squamous carcinoma vs Adenocarcinoma)	-0.690	0.502	0.245~1.026	0.059
PD-L1(%)	0.025	1.025	1.013~1.037	<0.001
TMB (muts/Mb)	0.152	1.165	1.082~1.254	<0.001
Neut(*10^9/L)	1.353	3.869	1.766~8.477	0.001
NLR	0.098	1.103	0.958~1.271	0.172

### 3.5 ROC for different indicators

**Figure 2:**
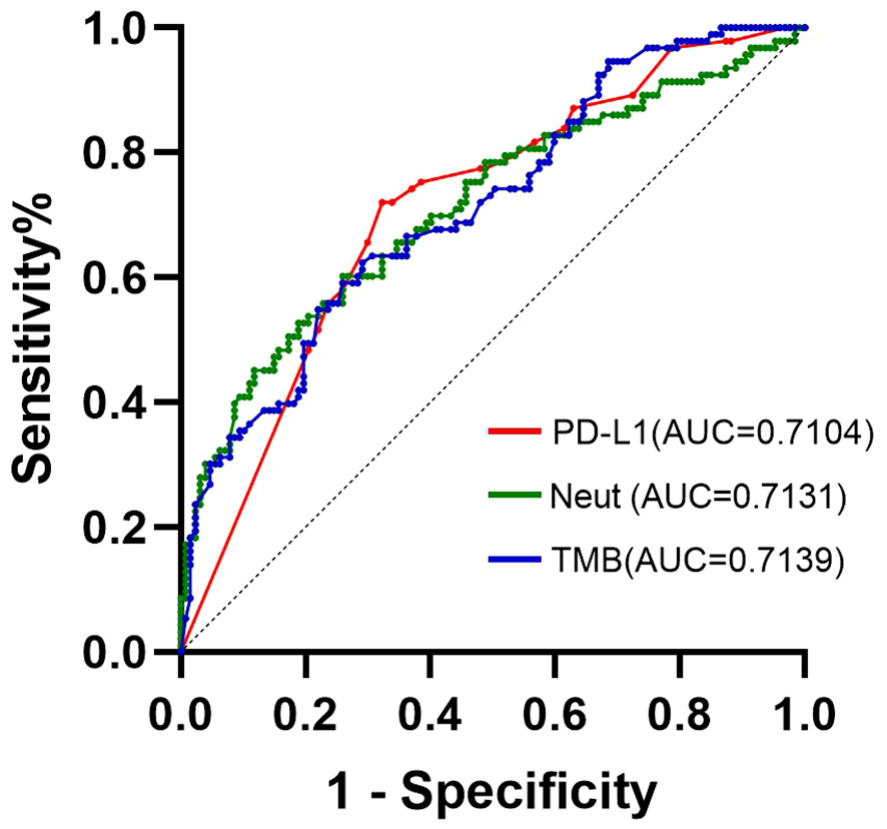
ROC curve of ORR group predicted by PD-L1, TMB, and neutrophils for patients with NSCLC.

According to the results of the multivariate analysis, the ROC curves of three independent prognostic factors, PD-L1, TMB, and neutrophils, of the ORR in patients with NSCLC were drawn. As shown in **Figure 2**, the AUC of the ROC curves of PD-L1, TMB, and neutrophils were 0.7104, 0.7139, and 0.7131 respectively, and were statistically significant (*P* < 0.001). When the sensitivity was 74.0% and the specificity was 61.2%, the best cut-off value for the Yoden index was 4.205 * 10 ^ 9/L.

### 3.6 Construction of nomogram prediction model

**Figure 3:**
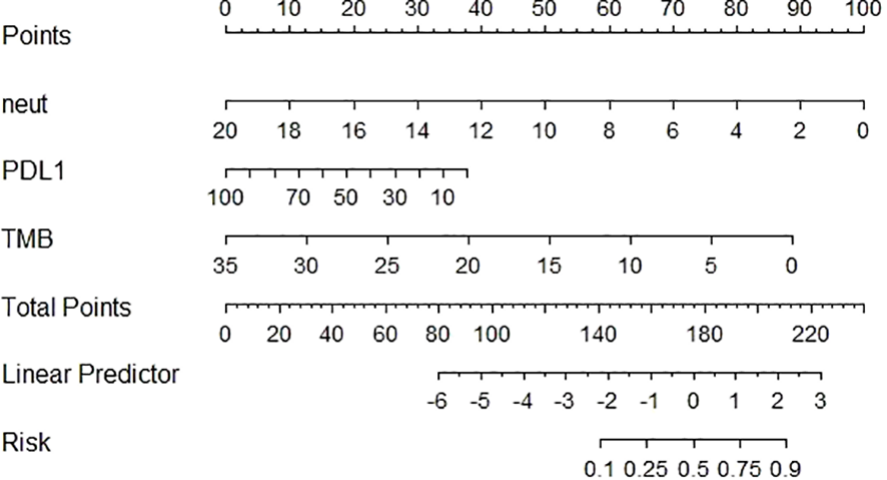
Construction of nomogram prediction model for NSCLC based on PD-L1, TMB, neutrophils.

**Figure 4:**
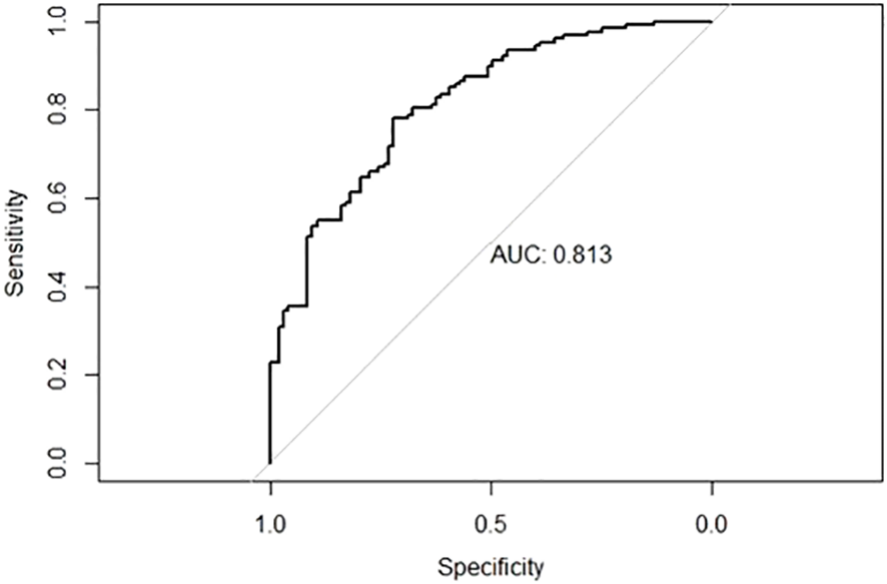
ROC curve for nomogram prediction model with AUC of 0.813.

According to the results of the ORR multivariate analysis and evaluation of the prediction efficiency of ROC, a line chart (nomogram) model based on PD-L1, TMB, and neutrophils was drawn using the nomogram function in the rms package, as shown in **Figure 3**. Neut expression range from 0*10^9/L to 20*10^9/L corresponds to the score threshold between 0 and 100 points, and PD-L1 expression range from 0% to 100% corresponds to the score threshold between 12 and 38 points. The TMB expression range ranges from 0muts/Mb to 35muts/Mb and the corresponding score threshold ranges from 0 to 88 points. Neutrophils and TMB had the widest range of risk scores and the most significant impact on prognosis. The points corresponding to the three indicators were added to the total points, which corresponded to the ORR risk value. The ROC curve drawn according to the model is shown in **Figure 4**. The AUC was 0.813, indicating medium accuracy. This shows that the model performs well in predicting the ORR in NSCLC.

### 3.7 The decision curve analysis

**Figure 5:**
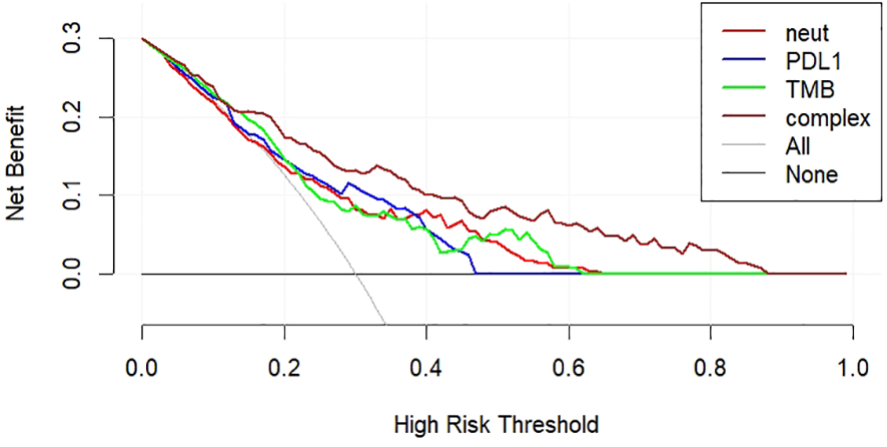
DCA curves for nomogram prediction models.

Among the factors of the nomogram prediction model, the DCA analysis of the complex model constructed using the three factors of “PD-L1,” “TMB,” and “neutrophils” and the simple model constructed using the three indexes individually is shown in **Figure 5**. The complex model had a higher net benefit ratio than the simple model for all thresholds in the range of 0.1–0.6.

## 4 Discussion

As tumors have entered the era of immunotherapy, both preclinical and clinical studies on the application of ICIs to patients with NSCLC have achieved good efficacy; however, with the wide application of ICIs in the clinic, especially for advanced NSCLC patients, only 15%–30% of patients can benefit from immunotherapy and survive for a long period of time, and even immune hyperprogression occurs (14). Therefore, there is an urgent need to identify suitable predictive biomarkers for precise tumor therapy. Extensively studied predictors of tumor immunotherapy include PD-L1, TMB, microsatellite instability (MSI), EGFR, and tumor-infiltrating lymphocytes (TILs). However, most of them have not been applied for detection before clinical immunization treatment, and based on the results of many studies, it is currently difficult for a single marker to achieve satisfactory predictive efficiency. The combination of multiple predictive markers to build a predictive model may be more effective in predicting the efficacy of ICIs.

The treatment regimen used in this study was a combination of anti-PD-1 ICIs and chemotherapy, which has become the standard first-line treatment for advanced NSCLC without EGFR mutations or high PD-L1 expression. Early studies (15) also showed that chemotherapy can improve tumor immunogenicity by inducing immunogenic cell death and new antigen release. Additionally both radiotherapy (16) and targeted therapy (17) have synergistic effects on ICIs. In the study of the correlation between short-term efficacy in patients with NSCLC and patient clinical characteristics, the results showed that male sex, age <60 years, history of smoking, and squamous carcinoma were the highest in the ORR group, and the differences were all statistically significant (*P* < 0.05). In addition, PD-L1, TMB, neutrophils, lymphocytes, NLR, and albumin were significantly correlated with ORR (*P* < 0.001). PD-L1 can be expressed on the surface of many types of tumor cells, and PD-L1 detected by immunohistochemistry (IHC) has become the first predictive biomarker for ICIs treatment approved by the Food and Drug Administration (FDA). Theoretically, the higher the expression level, the better the antitumor effect of ICIs. The results of this study showed that the mean value of PD-L1 was the highest in patients in the ORR group, which was also confirmed by many studies (18, 19). However, some studies (20) showed that even if ICIs were applied to patients with PD-L1 >50%, they performed poorer than chemotherapy. Bradley et al. (21) showed that patients with NSCLC with low PD-L1 expression had higher OS with ICIs than with chemotherapy. These studies have extended the application of ICIs to patients with low PD-L1 expression. Therefore, there is still great controversy regarding the criteria for PD-L1 expression in NSCLC. The second predictor included in this study was TMB. TMB represents the number of mutations per Megabyte (Mut/Mb) of DNA sequenced in a specific cancer (22), and the FDA approved a TMB score of ≥10 mut/Mb as the threshold for Pembrolizumab treatment of solid tumors, but the suitability of TMB-H needs to be demonstrated by further clinical studies. Similar to PD-L1, the higher the level of TMB expression in the tumor tissue, the more likely it is to benefit from immunotherapy. The results of this study showed that the mean value of TMB was the highest in the patients in the ORR group, as described previously. A number of studies (23, 24) have confirmed this. For example, Marabelle et al (25) studied the efficacy comparison between ICIs first-line immunotherapy and chemotherapy drugs in patients with advanced NSCLC, and found that ORR, PFS and OS in the immunotherapy group were relatively better than those in the chemotherapy group. But McGrail et al (26) studied the relationship between TMB and ICIs therapeutic efficacy in more than 10,000 solid tumors, and found that [for some tumors, such as breast cancer and prostate cancer, tumors with high TMB expression had lower ORR, which also indicated that TMB could not be completely used as a predictor of tumor immunotherapy. Future studies are needed to analyze the differences between different cancer types and establish TMB cutoff values in order to create a more standardized approach to the clinical use of TMB. Therefore, these limitations should be overcome before TMB can be widely used in the clinic, making TMB one of the promising predictive markers for immunotherapy.

Analysis of the correlation between PD-L1, TMB, and clinical features showed that there was a correlation between PD-L1 and EGFR, which was consistent with the results of Azuma et al. (27), whereas the results of Jiang et al. (28) clearly indicated that there was a negative correlation between EGFR mutation and PD-L1 expression. Therefore, it is suggested that expression of PD-L1 may be related to the mechanism of drug resistance to EGFR-TKIs. One study (29) found that in patients with NSCLC treated with EGFR-TKI, PD-L1-negative patients had a longer median progression-free survival (mPFS), which confirmed this idea. It has also been suggested (30) that EGFR-TKI treatment can downregulate PD-L1 expression and indirectly enhance antitumor immunity. Therefore, whether anti-PD-1/PD-L1 ICIs can be applied to EGFR-TKI-sensitive patients, especially in EGFR-TKI-resistant NSCLC patients, remains to be further studied. TMB grouping with 10 muts/Mb as the threshold correlated with sex, smoking history, drinking history, T stage, and EGFR. In a study of the relationship between tobacco and TMB, some experimental results showed that smoking can increase TMB (26, 31), and high TMB expression can increase the efficacy of immunotherapy, which explains why smokers are more sensitive to ICIs therapy. The results of this study also showed that more patients in the ORR group had a history of smoking. The results of this study showed a correlation between EGFR mutation type and TMB (*P* = 0.047). The results of this study (32) investigating the relationship between gene mutations and TMB. The results showed that low TMB expression was usually enriched in EGFR-mutant tumors, which is consistent with the results of this study. The results of the present study were inconsistent regarding the relationship between PD-L1 expression and TMB. The results of PD-L1 grouping with a 50% threshold and TMB numerical t-test showed a strong correlation (*P* = 0.004). However, there was no significant correlation between TMB grouping with 10 muts/Mb as the threshold and PD-L1 numerical t-test results (*P* = 0.937). Most studies have shown no significant correlation between them (20, 24, 33). Matthew et al. (24) showed that the progression-free survival time of first-line ICIs therapy was longer than that of chemotherapy in NSCLC patients with high TMB expression, regardless of PD-L1 expression level. This further suggests that the TMB is a beneficial biomarker for ICIs at all PD-L1 expression levels. Although this study showed no significant correlation between TMB and PD-L1 expression, both had similar predictive values. One study (32) investigated the efficacy of immunotherapy in NSCLC patients grouped according to TMB and PD-L1 expression; it found that patients with higher TMB and PD-L1 ≥50% had an ORR of up to 57% with anti-PD-1/PD-L1 ICIs, so the inclusion of TMB and PD-L1 expression in multivariate prediction models should yield great predictive power.

In the present study, the value of peripheral blood cells in predicting the short-term efficacy of immunotherapy was unexpected. Compared to those in the non-ORR group, the average values of neutrophils, lymphocytes, and NLR in the ORR group were relatively higher, and the differences were statistically significant. From the current studies on peripheral blood cells, most studies show that low neutrophil and high lymphocyte counts are positively correlated with the prognosis of tumors (34–37), which is not consistent with the results of this study. The high neutrophil count in this study positively correlated with short-term effects (*P* < 0.001). In humans, Neutrophils are the most abundant immune cells, accounting for 50%-70% of all white blood cells (38). Neutrophils participate in the composition of the tumor microenvironment and secrete chemokines that promote tumor proliferation, invasion, and angiogenesis, which are closely related to the occurrence and development of tumors. However, the number of neutrophils required to determine its effect on tumor immune efficacy is not completely convincing. A previous study (39) has proposed that the infiltrating neutrophils in the tumor have two sides; the phenotype of promoting tumor growth and metastasis, and the phenotype of inhibiting tumor growth. Moreover, the heterogeneity of tumor-associated neutrophils (TAN) was strong. According to phenotypic and functional differences, the most common neutrophils were mainly N1 and N2. N1 cells were anti-tumor neutrophils, while N2 cells showed the function of promoting tumor progression (40, 41). It has been proposed that infiltrating neutrophils in tumors can enhance their anti-tumor activity by recruiting immune cells (42). The role of neutrophil heterogeneity in immunotherapy has been widely studied (43). According to their surface marker molecules, some promote tumor growth, while others inhibit tumor progression. Therefore, for different neutrophil phenotypes, some studies targeting neutrophils have become the focus of research (44). Wang et al. (45) found that CD300ld expressed on neutrophils is a key immunosuppressive factor in the TME. Targeting CD3001d provides a new direction for tumor immunotherapy. Most studies have focused on the measurement of neutrophils, lymphocytes, and NLR before immunotherapy. Immunotherapy can also induce neutrophil responses and increase the number of neutrophils infiltrating tumors. Therefore, it is important to infer the dynamic changes in neutrophil numbers to predict the efficacy of immunotherapy. Most studies have investigated the relationship between neutrophils and the long-term prognosis of ICIs treatment, very few studies have investigated their relationship with the short-term efficacy of ICIs has. Moreover, studies (46) have shown that neutrophils can stimulate T cell response and increase anti-tumor response in the early stage of immunotherapy. In addition, there exists a heterogeneity of neutrophils and complex responses in the tumor immune microenvironment. Thus, more studies are needed to confirm the role of neutrophils in immunotherapy and to conclusively demonstrate that neutrophils are positively correlated with immunologic efficacy.

In this study, through univariate and multivariate analyses of ORR, it was determined that PD-L1, TMB, and neutrophils were independent prognostic factors of ORR in patients with NSCLC, and the AUC values of the ROC curves based on the three indices were 0.7104, 0.7139, and 0.7131, respectively, indicating that the three have good predictive efficiency. The line chart (nomogram) model drawn according to these three indicators showed good predictive ability, and the results showed that neutrophils and TMB had the widest range of risk scores and the most significant impact on prognosis. The AUC value under the ROC curve for the predictive model was 0.813. According to the DCA curve, the net return of the prediction model constructed using the three indicators was higher than that of the single-index prediction model, which again proved its prediction efficiency. From these results, it can be seen that the nomogram model constructed by the expression level of PD-L1 and TMB combined with other characteristics has a good predictive effect on the immune efficacy of patients with advanced NSCLC. Sun et al. (47) constructed a classification model based on PD-L1 and TILs and found that this model was significant for the choice of ICI treatment. This study provides feasibility for the predictive efficacy of PD-L1, TMB and neutrophil models in NSCLC patients. But few studies have been conducted on multiple variables to construct an ICI predictive model. Therefore, we need to study more predictive factors, build a more comprehensive prognostic model to predict the prognosis of patients with NSCLC, and formulate more individualized treatment strategies.

The current study has a few limitations. Firstly, the results of PD-L1 and TMB testing were affected by different testing platforms and the lack of uniform standards for diagnostic results. In addition, the heterogeneity of PD-L1 and TMB expression affected the test results. Furthermore, differences were noted in the PD-L1 expression levels between primary lung lesions and metastatic brain tissues in NSCLC patients. Regarding the detection of TMB, some studies (48) have shown that the detection success rate of TMB in peripheral blood is higher than that in tumor tissue TMB; however, this conclusion needs to be further confirmed. Secondly, many factors influence the routine clinical blood index, which cannot guarantee the accuracy of its influence on the results. Finally, this was a single-center, retrospective study with a relatively small sample size.

## 5 Conclusion

PD-L1, TMB, and neutrophils are prognostic factors for the short-term efficacy of anti-PD-1 combined chemotherapy in NSCLC. A predictive model constructed using these three indicators could further improve the predictive efficiency of ICIs efficacy.

## Data availability statement

The original contributions presented in the study are included in the article/supplementary material. Further inquiries can be directed to the corresponding author.

## Ethics statement

The studies involving humans were approved by the Ethics Committee of the First Affiliated Hospital of Zhengzhou University. Ethical number: 2023-KY-1063-001. The studies were conducted in accordance with the local legislation and institutional requirements. The participants provided their written informed consent to participate in this study. Written informed consent was obtained from the individual(s) for the publication of any potentially identifiable images or data included in this article.

## Author contributions

SS: Writing – original draft. YW: Writing – original draft. YL: Writing – review & editing. YY: Conceptualization, Writing – review & editing. JK: Data curation, Writing – review & editing. SG: Formal analysis, Writing – review & editing. HC: Investigation, Writing – review & editing. LH: Methodology, Writing – review & editing. XS: Project administration, Writing – review & editing. ZL: Software, Writing – review & editing. TL: Writing – review & editing, Resources. JW: Resources, Writing – review & editing. BZ: Supervision, Writing – review & editing. PL: Validation, Writing – review & editing. YZ: Visualization, Writing – review & editing. DY: Supervision, Writing – review & editing.
